# Sinonasal/basicranial myxofibrosarcoma: a report of 6 surgical cases combined with a literature review

**DOI:** 10.1016/j.bjorl.2022.02.001

**Published:** 2022-02-15

**Authors:** Hongbing Li, Huan Wang, Dehui Wang

**Affiliations:** Eye and ENT Hospital of Fudan University, Department of Otolaryngology, Shanghai, China

**Keywords:** Myxofibrosarcoma, Surgery, Prognosis

## Abstract

**Objective:**

The aim of this study was to report 6 cases of sinonasal/basicranial myxofibrosarcoma and review demographic data, clinical symptoms, diagnostic methods, therapeutic methods, and prognosis on this subject in the literature.

**Methods:**

We conducted a retrospective analysis of six patients referred to our hospital during a 10-year period from 2010 to 2020.

**Results:**

In this series, there were four males and two females. The tumours originated from the maxillary sinus in all six cases. The symptoms were nonspecific, and all patients underwent endoscopic resection alone or combined with open resection. The postoperative period was uneventful. All patients were diagnosed with myxofibrosarcoma. After a median follow-up period of 36 (6–52) months, the recurrence rate of MFS was 66.7% (4/6), and the mortality rate was 50% (3/6).

**Conclusion:**

Sinonasal/basicranial MFS is a rare neoplasm, and the most common primary site is the maxillary sinus. When diagnosing MFS, osteolysis may help rule out benign lesions. So far surgery is the mainstay of treatment for sinonasal/basicranial MFS. Our surgical strategy using endoscopic resection or endoscopic resection combined with open resection to achieve a negative surgical margin and using re-excision operations to treat relapsed cases may be recommended to rhinologists who treat MFS. Prospective randomised trials are needed to study the role of chemotherapy, radiotherapy, and our surgical strategy for sinonasal/basicranial MFS. Diagnosing patients at an earlier stage and better patient compliance with follow-up plans may improve the prognosis of patients.

## Highlights

• So far surgery is the mainstay of treatment for sinonasal/basicranial MFS.
• Negative surgical margin and re-excision for relapsed cases may be recommended to rhinologists.
• Early diagnosis and better patient compliance may improve the prognosis.

## Introduction

Myxofibrosarcoma (MFS) is a myxoid variant of malignant fibrous histiocytoma, and it also is a malignant fibroblast tumour with a matrix of myxoid, visible arc-like vessels, and tumour cells showing varying degrees of atypia. MFS is the most common soft tissue sarcoma that appears in late adult life and is typically a low-grade malignancy. MFS is mainly encountered in the lower extremities (77%), trunk (12%), and retroperitoneum or mediastinum (8%), and only a few corresponding cases in the head and neck region have been reported to date, with 3%–10% involvement.^1^ Willems and colleagues^2^ demonstrated that most cases have complex cytogenetic anomalies, and such alterations were observed in tumours of all grades. Furthermore, this group observed that the tumours that locally recurred had more complex cytogenetic aberrations when compared with that of tumours that did not recur. Many novel molecular markers, such as Integrin-10, mesenchymal epithelial transition factor, NF1 gene, and ezrin, are reported to be related to the mechanisms of tumorigenesis of MFS.^3^ However, the molecular pathogenesis of MFS is not well-understood. Mentzel et al.^4^ divided the lesions into low, intermediate, and high grades to simplify the Merck classification system.

The final diagnosis of a MFS is based on the microscopic, immunohistochemical, and ultrastructural features of this lesion. Light-microscopic features include the (1) Presence of alternating hypocellular, myxoid areas, and hypercellular, fibrous areas; (2) Spindle and stellate cells in myxoid matrix; (3) Aggregation of neoplastic cells or inflammatory cells; (4) Curvilinear, thin-walled blood vessels prominent in the myxoid areas; and (5) Pleomorphic nuclei.^5^, ^6^ Immunologic staining is typically positive for vimentin and CD34, which indicates the tumour’s fibroblastic origin; they are negative for S100 protein.^6^ When a biopsy specimen causes suspicion of MFS, definite diagnosis is difficult and radiological images show features of malignant tumour, complete resection of the mass lesion for definite diagnosis and curative treatment is feasible. Overall Survival (OS) ranges from 61% to 77% at 5-year and Local Recurrence (LR) rates range from 16% to 57%.^7^

There are few reports in the literature regarding the characteristics of MFS in the sinonasal/basicranial regions and the subsequent survival time. Hence, the purpose of the present study was to report clinical cases of MFS from our hospital and review the epidemiology, clinical presentation, diagnosis, treatment, and prognosis of patients with sinonasal/basicranial MFS.

## Methods

We retrospectively collected data for all patients who presented with MFS involving the nasal cavity, paranasal sinuses (sphenoid, maxillary, ethmoid, and frontal sinuses), and skull base. The cases were retrieved from (Medical Institutions), during a 10-year period from 2010 to 2020. The patients’ medical records were analysed for demographic data, location, previous surgical history, clinical symptoms, pathological features, therapeutic methods, relapse, and survival. Postoperatively, MFS patients were observed using enhanced MRI for tumour recurrence. The follow-up time plan was every three months in the first year after the operation, every six months from the second year after the operation, and every year from the sixth year after the operation. The extent of surgical resection was obtained by comparing preoperative and postoperative enhanced MRI. Follow-up time was defined as the interval between the last date of the initial treatment and the time of death or last follow-up. This study was approved by the Institutional Review Board of (Medical Institutions). All patients provided informed consent regarding the use of clinical information and photographs for research (Table 1).

**Table 1:** Features of 6 cases with sinonasal/basicranial myxofibrosarcoma.

Patient/sex/age	Lateral/Origin site	Extent site	Main sympton	TNM stage	Pathological grade	Previous operation times, n	CT findings osteolysis	Surgical procedure	Adjuvant therapy	Recurrence	Follow-up (months)	Outcome
1/M/44	L/MS	OW, MS, AB, ES, PP, ZA, PF	Proptosis	T2N0M0	low	0	Yes	ESR, ME, OE	RC CT	1	52	DOD
2/F/51	L/MS	NC, MS, ES, PN, NP	Nasal obstruction epistaxis	T2N0M0	middle	0	Yes	ESR	RT	4	49	DOD
3/F/62	R/MS	Cheek, NC, ES, PN, PF, IF, OW, AB, HP	Facial swelling	T2N0M0	high	0	Yes	ESR	NO	3	7	DOD
4/M/30	L/MS, NC	NC, MS, cheek, NDDA, IF, OW, ES, APN	Nasal obstruction epistaxis	T2N0M0	low	2	Yes	ESR, MME, CLO	RT	4	46	NED
5/M/45	L/MS	MS, IM, AB, cheek, HP	Swollen upper jaw gums	T2N0M0	low	0	Yes	ESR, CLO	NO	NO	28	NED
6/M/12	L/MS	MS, HP, AB, cheek, SNC, MIM, IF	Facial swelling	T2N0M0	low	0	Yes	ESR, ME, CLO	RT	NO	6	NED

M, male; F, female; L, left; R, right; OW, orbital wall; MS, maxillary sinus; AB, alveolar bone; ES, ethmoid sinus; PP, pterygoid process; ZA, zygomatic arch; PF, pterygopalatine fossa; NC, nasal cavity; PN, posterior nostril; NP, nasopharynx; IF, infratemporal fossa; HP, Hard palate; NDDA, nasolacrimal duct dacryocyst area; APN, anterior and posterior nostrils; IM,inferior meatus; SNC, side of nasal cavity; middle and inferior meatus; ESR, Endoscopic surgical resection; ME, maxillary excision; MME, most ME; OE, orbital exenteration; CLO, Caldwell-Luc operation; RT, radiotherapy; CT, chemotherapy; NED, no evidence of disease; DOD, die of disease.

## Results

### Demographic data

A summary of the patients in this series is shown in Table 1. Of the six patients identified, four were male and two were female. The age at the time of surgery ranged from 12 to 62 years, with a median age of 44.5 years. The tumours originated in the maxillary sinus in all six cases. The symptoms were nonspecific, and the main symptoms included nasal obstruction and epistaxis, facial numbness and pain, facial swelling, proptosis, diplopia, and swollen upper jaw gums. Enhanced CT for patients showed soft tissue masses with uneven density, unclear boundaries, and uneven enhancements. Further, it showed osteolysis in all six patients. Enhanced MRI in patients showed a low and medium signal on T1WI, an uneven high and low signal on T2WI, and an uneven enhancement. According to the American Joint Committee on Cancer TNM classification system (7th Edition, 2010),^8^ the tumour grades of all six patients were T2N0M0.

**Figure 1:**
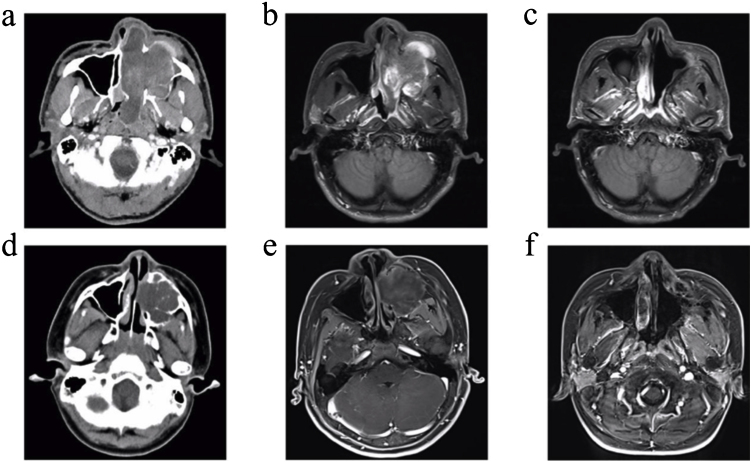
Images of patient 4 and patient 6. (a) Enhanced CT for patient 4 showed a large soft-tissue mass in the left maxillary sinus and nasal cavity that invaded the cheek, nasolacrimal duct dacryocyst area, infratemporal fossa, orbital wall, ethmoid sinus, anterior and posterior nostrils, and partly destroyed the bone of the sinus cavity. (d) Enhanced CT for patient 6 showed a large expansive soft-tissue mass in the left maxillary sinus that invaded the hard palate, nasal cavity, alveolar bone, inferior turbinate, and partly destroyed the bone of the sinus cavity. (b) Enhanced MRI for patient 4 and (e) enhanced MRI for patient 6 showed uneven enhancement of the masses. (c) Enhanced MRI for patient 4 and (f) enhanced MRI for patient 6 revealed no masses in the surgical cavity.

In the present study, patient 4 presented with a large soft-tissue mass in the left maxillary sinus that invaded the cheek, nasolacrimal duct dacryocyst area, infratemporal fossa, orbital wall, ethmoid sinus, nasal cavity, and anterior and posterior nostrils. Patient 6, a 12-year-old boy, presented with a large expansive soft-tissue mass in the left maxillary sinus that invaded the hard palate, alveolar bone, cheek, side wall of the nasal cavity, middle and inferior nasal meatus, and infratemporal fossa. Preoperative images from enhanced CT and enhanced MRI for patients 4 and 6 are shown in Fig. 1 (a, b, d and e). Patient 4 underwent a complete resection of the tumours via endoscopic resection combined with a maxillary excision with the Caldwell-Luc approach. Patient 6 underwent complete resection of the tumours via endoscopic resection combined with maxillectomy via Caldwell-Luc operation. Later, Patient 4 underwent four additional operations due to four instances of tumour recurrence. After 46-months, through enhanced MRI examination, the patient was found to be in a tumour-free state (Fig. 1c). After six months recovery for patient 6, there was no recurrence based on enhanced MRI imaging examinations (Fig. 1f).

### Treatment and outcomes

All patients underwent endoscopic resection or endoscopic resection combined with open resection. Open resection included maxillary excision using the Weber-Fergusson approach, orbital exenteration, and the Caldwell-Luc operation. Four cases of MFS were treated with adjuvant radiotherapy after surgery, and patient 1 was managed with chemotherapy. The tumours in all six patients were completely resected at our institution during the initial surgery. Only patient 5 experienced postoperative epistaxis. No surgery-related complications were observed in the other patients. The final diagnosis was based on pathological findings. All six patients were diagnosed with MFS. After a median follow-up period of 36 (6–52) months, no patient was lost to follow-up. Four patients had postoperative recurrence; the recurrence rate of MFS was 66.7% (4/6). Three patients (50%) died due to tumour progression. Patient 2 underwent three more surgical resections, patient 3 underwent two more surgical resections, patient 4 underwent four more surgical resections, and patient 1 did not undergo reoperation due to high risk but did receive further radiotherapy and chemotherapy. No patient had lymph node metastasis or distant metastasis.

## Discussion

We conducted a comprehensive MEDLINE search for all cases of sinonasal/basicranial MFS, and only 10 articles were identified (Table 2).^1^, ^9^, ^10^, ^11^, ^12^, ^13^, ^14^, ^15^, ^16^, ^17^ The earliest report of the disease was a single case report published in 1982.^9^ Including our six patients, a total of 16 cases have been reported. The number of cases we reported is the largest to our knowledge, and other studies are single case reports. There were 10 male and six female patients. The ages of patients in these reports ranged from 12 to 69 years, and the median age was 50.5 years. The most common primary site was the maxillary sinus (12/16), and there was one case each with the sphenoid sinus, pterygopalatine fossa, pterygopalatine fossa, maxilla, and nasal cavity as the primary site. In our series, all six cases stemmed from the maxillary sinus and is the largest reported case series of MFS in the maxillary sinus. The symptoms were nonspecific, and the main symptoms are shown in Table 1, Table 2. For head and neck sarcomas, a previous study with 11,481 adult cases has reported the most common histologic subtypes were malignant fibrous histiocytoma (MFH, n = 3563, 31%), Kaposi sarcoma (KS, n = 2311, 20%), and hemangiosarcoma (n = 895, 8%); the male gender predominated, with 8330 cases (73%) reported; females accounted for only 3151 cases (27%);the median age group affected was 55–59 years; the most common primary site was the skin and soft tissues (n = 7244, 63%), followed by the bones of the skull and face (n = 1277, 11%) and the oral cavity (n = 1108, 10%).^18^ Kalavrezos et al. reported that early diagnosis of head and neck sarcomas remains a challenge due to non-specific symptomatology.^19^

**Table 2:** Reported cases of myxofibrosarcoma in the sinonasal/basicranial region.

Patient	Ref.	Sex/Age	Lateral/Origin site	Size, cm	Symptoms	CT findings osteolysis	Treatment procedure	Follow-up period
1	Pomerantz et al.,^9^ 1982	M/58	R/MS	7	Gums swelling	Yes	Surgery	Unspecified
2	Lam et al.^11^ 2002	M/55	Bilateral/SS	NR	Blood-stained rhinorrhoea	Yes	Surgery	8-months, free of disease
3	Enoz et al.^17^ 2007	F/36	L/MS	5.5	Facial swelling	Yes	Surgery	7-months, free of disease
4	Norval et al.^12^ 2011	M/69	L/MS	5	Facial pain	Yes	Radiotherapy, Chemotherapy	Died after disease, 1-year later
5	Krishnamurthy et al.,^13^ 2011	F/42	L/IF	5.3	Swelling below earlobe	Yes	Surgery, Radiotherapy	26-months, free of disease
6	Nakahara, et al.,^14^ 2012	M/52	R/Maxilla	5.5	Cheek discomfort	Yes	Surgery, Radiotherapy	20-months, free of disease
7	Taghi et al.^15^ 2012	F/50	L/MS	NR	Chronic sinus pain	Yes	Surgery, Radiotherapy	6-months, free of disease
8	Cante et al.^16^ 2013	M/66	L/MS	NR	Facial pain	Yes	Radiotherapy, Chemotherapy	6-months, complete remission of disease
9	Dell'Aversana Orabona et al.^1^ 2014	M/35	R/PF	8.4	Facial swelling	Yes	Surgery, Radiotherapy	27-months, free of disease
10	Wong et al.^10^ 2017	F/61	L/MS	NR	Eye proptosis	Yes	Surgery, Radiotherapy	Unspecified

M,male; F, female; R, right; L, left; NR, not report; MS, maxillary sinus; SS, sphenoid sinus; IF, infratemporal fossa; PF, pterygopalatine fossa.

Prior to pathological diagnosis, the diagnosis of MFS in the sinonasal regions is also challenging because symptoms are not characteristic, and radiologic findings are usually nonspecific. CT generally shows bony alterations and expansive or destructive type changes. Benign tumours, such as pleomorphic adenoma, usually present with well-defined tissue masses and expansile bony changes. Osteolysis is an indirect sign of malignancy. In this series and other cases reported in the sinonasal/basicranial regions (Table 1, Table 2), the proportion of MFS cases with osteolysis presentation was 100%. Therefore, when diagnosing MFS, osteolysis may help rule out benign lesions. Compared to CT, MRI can more clearly show the extent of malignant tumours. Compared with the Apparent Diffusion Coefficient (ADC) value of non-myxoid tumours, that of mucinous tumours is obviously high, and DWI MR imaging has been shown to be helpful in assessing the composition of tumour cells in soft tissue sarcomas.^20^ The idea using a standardized MR program to compare the ADC values of various types of sarcomas may provide a new way for researchers to diagnose MFS through MRI in the future (Table 2).

Surgery is the primary and most effective treatment for MFS.^21^ To prevent LR, margin-negative surgical resection is the cornerstone of treatment for patients with MFS. Therefore, margin-negative surgical resection should be the aim of a rhinologist. Intraoperative margin assessment with frozen sections is notoriously difficult and unreliable. An experienced rhinologist who can distinguish normal tissue from tumour tissue is necessary. In order to treat sinonasal/basicranial MFS, the surgical procedures included open and endoscopic resection. Open resection includes maxillectomy with the Weber-Fergusson approach, maxillectomy via the midfacial degloving approach, orbital exenteration, Caldwell-Luc operation.^9^, ^10^, ^15^ The excised portion can be reconstructed using a titanium plate, a free rectus abdominis myocutaneous flap, an artificial denture, or a microvascular free flap.^1^, ^9^, ^10^, ^14^ In our series, we used endoscopic resection or endoscopic resection combined with open resection to achieve a negative surgical margin. The use of endoscopy provides a broad surgical field and excellent visibility, thereby avoiding surgical morbidity. Endoscopy also prevents induction of blindness and destruction of adjacent structures. In addition, we didn’t perform maxillary resection with the Weber-Fergusson approach except for the tumor invading the skin of the cheek, which demonstrated the advantages of endoscopic resection combined with Caldwell-Luc method for minimal invasiveness, and more desirable cosmetic appearance. Only patient 5 experienced postoperative epistaxis, and the other patients had no obvious postoperative complications. In addition, patient 5 had the a 28-month recurrence-free interval, which was the longest among those reported for cases involving the sinonasal/basicranial regions in the MFS literature. We also used re-excision operations to treat the relapsed cases. Patient 4 underwent four additional operations due to four instances of tumour recurrence. Currently, the patient remains in a tumour-free state and has the longest tumour-free survival in all of the sinonasal/basicranial MFS literature. Dell'Aversana Orabona et al.^1^ also proposed that re-excision of recurrent lesions is a way to enhance survival. So, our surgical strategy may be recommended to rhinologists who treats MFS. Although our surgical strategy may be beneficial in patients with sinonasal/basicranial MFS, a large prospective study will be needed to further delineate our findings. For head and neck sarcomas, adequate clearance of locoregional disease and prevention of distant micrometastases are key to improved disease-free survival outcomes. A recent study has reported that the treatment for most bone sarcomas is neoadjuvant chemotherapy followed by compartmental resection; for soft tissue sarcomas of the head and neck, treatment is complex and depends on grade: surgery is the principal mode of treatment in low-grade tumours that are amenable to resection; high-grade tumours can be treated with neoadjuvant chemotherapy followed by surgery and radiotherapy.^19^ However, for sinonasal/basicranial MFS, the role of chemotherapy and radiotherapy is unclear. Prospective randomized trials are needed to study the role of chemotherapy and radiotherapy. However, because of the low side effects of local treatment and ability to prevent tumour recurrence in soft tissue sarcomas,^22^ radiotherapy is recommended for MFS patients to prevent postoperative LR and distant metastasis.^1^, ^10^, ^14^ Cante et al.^16^ reported that MFS patients in their hospital remained in complete unmaintained clinical and instrumental complete remissions for 18-months after combined chemoradiation, with no late side effects. Therefore, we recommend palliative chemoradiotherapy for MFS patients who cannot be treated surgically. For head and neck sarcomas, Kevin et al.^18^ reported that Cause-specific 2-, 5-, and 10-year survival rates were 76%, 66%, and 61% for adults and male gender, absence of radiation therapy, and stage I disease were associated with improved cause-specific survival reaching statistical significance. James et al.^23^ reported that positive margins, large tumors, and high-grade histology continue to have an impact on local control and overall survival. For patients with MFS in any part of the body, OS ranges from 61% to 77% at 5-year and LR rates range from 16% to 57%.^7^ Resection margin status at the initial operation remains the most important factor associated with survival and LR.^21^ Furthermore, Haglund et al.^5^ demonstrated reduced LR rates for patients with wider microscopic margins. Among patients with negative margins, six of 15 patients with margins <1 cm had an LR, whereas none of the patients with margins of at least 1 cm had an LR. In our series of MFS of the sinonasal/basicranial region, patients had a higher LR rate of 67% and a lower OS of 50% with a median follow-up period of 36-months. The reason may because a wider surgical resection margin is challenging to achieve, and the margin-negative surgical resection rates are lower in the delicate sinonasal/basicranial region. When going to the hospital for diagnosis with symptoms, most cases have been stage T2,^9^, ^13^, ^17^ which leads to large surgical trauma and difficult margin-negative surgical resection. For margin-negative surgical resection, many patients require maxillary resection, and orbital exenteration is required for lesions involving the eyeball.^9^, ^10^, ^17^ Therefore, it is necessary to diagnose patients at an early stage, which can be facilitated by finding some MFS tumour markers. No patient had lymph node metastasis or distant metastasis in the sinonasal/basicranial regions. Therefore, we believe that re-excision of recurrent lesions may significantly enhance survival in patients with MFS in the sinonasal/basicranial regions compared to that of patients with MFS in other parts. However, the role of re-excision operations in relapsed cases depends on regular follow-up. For example, patient 1 did not adhere to the follow-up time specified by us. As a result, at the first visit after the operation, the tumour had recurred extensively, transferred to the skull base, and was not resectable. Therefore, we should strive to improve patient compliance.

## Conclusion

Sinonasal/basicranial MFS is a rare neoplasm, and the most common primary site is the maxillary sinus. When diagnosing MFS, osteolysis may help rule out benign lesions. So far surgery is the mainstay of treatment for sinonasal/basicranial MFS. Our surgical strategy using endoscopic resection or endoscopic resection combined with open resection to achieve a negative surgical margin and using re-excision operations to treat relapsed cases may be recommended to rhinologists who treat MFS. Prospective randomized trials are needed to study the role of chemotherapy, radiotherapy, and our surgical strategy for sinonasal/basicranial MFS. Diagnosing patients at an earlier stage and better patient compliance with follow-up plans may improve the prognosis of patients.

## Author contributions

Hongbing Li and Huan Wang were involved in the follow-up of patients and data collection. Hongbing Li and Huan Wang processed the data, performed the analysis, and participated in writing of the manuscript and designing of the figures and tables. Dehui Wang was involved in planning and supervising the work and in the final approval of the manuscript. All authors discussed the results and critically commented on the manuscript. Hongbing Li and Huan Wang contributed equally to this study.

## Funding

This work was supported by the Shanghai Shenkang Hospital Development Center/New Frontier Technology Joint Research Program (Project number SHDC12018118).

## Conflicts of interest

The authors declare no conflicts of interest.
